# Isometamidium chloride and homidium chloride fail to cure mice infected with Ethiopian *Trypanosoma evansi* type A and B

**DOI:** 10.1371/journal.pntd.0006790

**Published:** 2018-09-12

**Authors:** Gebrekrustos Mekonnen, Elmi Fahiye Mohammed, Weldu Kidane, Awol Nesibu, Hagos Yohannes, Nick Van Reet, Philippe Büscher, Hadush Birhanu

**Affiliations:** 1 College of Veterinary Medicine, Mekelle University, Mekelle, Ethiopia; 2 College of Veterinary Medicine, Samara University, Afar, Ethiopia; 3 IGAD Sheikh Technical Veterinary School (ISTVS), Nairobi, Kenya; 4 School of Veterinary Medicine, Wollo University, Dessie, Ethiopia; 5 Institute of Tropical Medicine, Nationalesstraat 155, 2000 Antwerp, Belgium; Hunter College, CUNY, UNITED STATES

## Abstract

**Background:**

*Trypanosoma evansi* is mechanically transmitted by biting flies and affects camels, equines, and other domestic and wild animals in which it causes a disease called surra. At least two types of *Trypanosoma evansi* circulate in Ethiopia: type A, which is present in Africa, Latin America and Asia, and type B, which is prevalent in Eastern Africa. Currently, no information is available about the drug sensitivity of any Ethiopian *T*. *evansi* type.

**Methodology/principal findings:**

This study was conducted with the objective of determining the *in vivo* drug sensitivity of two *T*. *evansi* type A and two type B stocks that were isolated from camels from the Tigray and Afar regions of Northern Ethiopia. We investigated the efficacy of four trypanocidal drugs to cure *T*. *evansi* infected mice: melarsamine hydrochloride (Cymelarsan), diminazene diaceturate (Veriben and Sequzene), isometamidium chloride (Veridium) and homidium chloride (Bovidium). Per experimental group, 6 mice were inoculated intraperitoneally with trypanosomes, treated at first peak parasitemia by daily drug injections for 4 consecutive days and followed-up for 60 days. Cymelarsan at 2 mg/kg and Veriben at 20 mg/kg cured all mice infected with any *T*. *evansi* stock, while Sequzene at 20 mg/kg caused relapses in all *T*. *evansi* stocks. In contrast, Veridium and Bovidium at 1 mg/kg failed to cure any *T*. *evansi* infection in mice.

**Conclusions/significance:**

We conclude that mice infected with Ethiopian *T*. *evansi* can be cured with Cymelarsan and Veriben regardless of *T*. *evansi* type. In contrast, Veridium and Bovidium are not efficacious to cure any *T*. *evansi* type. Although innate resistance to phenanthridines was previously described for *T*. *evansi* type A, this report is the first study to show that this phenomenom also occurs in *T*. *evansi* type B infections.

## Author summary

Surra is a vector borne disease in camels, horses, water buffaloes, cattle and other domestic animals caused by *Trypanosoma (T*.*) evansi*. This protozoan parasite is transmitted by biting flies such as tabanids and stable flies and is endemic in many countries in Northern and Eastern Africa, Latin America and Asia. Surra is responsible for high economic losses due to mortality and morbidity of draught animals and leads to animal trade restrictions in endemic regions. Control of surra is mainly based on the treatment of sick animals presenting clinical symptoms. In Ethiopia two different types of *T*. *evansi* (A and B) have been described, yet no data existed about the drug sensitivity of any *T*. *evansi* type. In this study, we show for the first time that *T*. *evansi* type B is naturally *in vivo* resistant to the phenanthridine class of trypanocidal drugs, a phenonomen that was previously described for *T*. *evansi* type A. All Ethiopian *T*. *evansi* types are sensitive to melarsamine hydrochloride and diminazene diaceturate. Unfortunately, the most efficacious drugs are either not registered in Ethiopia or escape quality control of the active substance in commercial drug formulations. Furthermore, the inefficacious drugs remain accessible on the market despite their toxicity for animals.

## Introduction

African trypanosomoses (AT) are neglected parasitic diseases of humans and animals caused by various subgenera of pathogenic trypanosomes (Trypanozoon, Dutonella and Nannomonas). While human African trypanosomosis (HAT) has reached the point where elimination is being envisaged, animal African trypanosomosis (AAT) is still one of the major parasitic disease constraints to animal productivity in sub-Saharan Africa causing an estimated annual loss between 0.7 and 4.5 billion USD [1–4]. In Ethiopia, AAT has been described as a major impediment to livestock development and agricultural production, contributing negatively to development in general and to food self-reliance efforts of the country in particular. Both tsetse-transmitted (TTAT) and non tsetse-transmitted African trypanosomiasis (NTTAT) are endemic to the country. TTAT are due to *Trypanosoma (T)*. *congolense*, *T*. *vivax*, and *T*. *brucei brucei*, whereas NTTAT are due to mechanically transmitted *T*. *evansi* and *T*. *vivax*, and the sexually transmitted *T*. *equiperdum* [5–12].

Surra is the number one protozoan disease of camels and is caused by *T*. *evansi*. Infected camels and equines may die within 3 months after onset of the disease. Moreover, cattle, water buffalo, pigs, goat and sheep infected with *T*. *evansi* suffer from immunosuppression, resulting in increased susceptibility to other diseases and vaccination failure against classical swine fever and *Pasteurella multocida* [13–15]. The distribution of the disease mainly coincides with that of camels in the semi-desert areas of the country [5,7,16,17].

The control of surra relies mainly on the use of the trypanocidal drugs: the diamidine diminazene diaceturate, phenanthridines such as homidium salts (homidium chloride and homidium bromide) and isometamidium chloride, and the arsenical melarsamine hydrochloride [18–22]. Isometamidium chloride is mainly used as a prophylactic drug and provides on average 3 months protection against trypanosome infection. Homidium salts have limited prophylactic properties and are mainly used as therapeutic agent [23]. Diminazene diaceturate and melarsamine hydrochloride are exclusively used as therapeutic agents [24].

Control of AT through chemotherapeutics is challenged by the emergence of drug resistance [25,26]. Resistance of *T*. *congolense* to isometamidium treatment has been reported in various areas of Ethiopia [11,12,27]. Similarly, Hagos and co-workers reported on the resistance of *T*. *equiperdum* against diminazene diaceturate [28]. Till present, there is no published evidence for drug resistance in Ethiopian *T*. *evansi*. However, isometamidium treatment failures in *T*. *evansi* infections have been documented in Sudan, China, the Phillipines and Venezuela [22,29–33]. Ethiopian *T*. *evansi* stocks are composed of at least two types that are grouped into *T*. *evansi* type A and *T*. *evansi* type B based on the restriction enzyme profile of the kDNA minicircles [34,35]. *T*. *evansi* isolates with minicircle type A usually have the RoTat 1.2 variable surface glycoprotein (VSG) and are the most abundant in East and West Africa, Latin America and Asia [5,35–39]. In contrast, *T*. *evansi* type B is less common and so far has only been isolated from camels in Chad, Kenya and Ethiopia [5,34,35,39–41]. In a former study, we isolated *T*. *evansi* type A and type B stocks from camels in the Afar and Tigray regions in Northern Ethiopia [5,41]. The present study was undertaken to investigate the *in vivo* drug sensitivity profiles of some of these *T*. *evansi* stocks in mice with regard to diminazene diaceturate, isometamidium chloride, homidium chloride and melarsamine hydrochloride.

## Materials and methods

### Ethical considerations

Handling and use of experimental mice was approved by the College of Veterinary Medicine, Mekelle University (CVM-CRC/21/08), in line with the National Research Ethics Review Guideline of the Ethiopian Ministry of Science and Technology, Addis Ababa, 2014.

### *T*. *evansi* stocks

For this study, we used two *T*. *evansi* type A (MCAM/ET/2013/004 and MCAM/ET/2013/009) and two *T*. *evansi* type B (MCAM/ET/2013/010 and MCAM/ET/2013/014) stocks, that we previously isolated from dromedary camel in Tigray and Afar, Northern Ethiopia [41]. All four stocks were typed as dyskinetoplastic trypanosomes based on absence of amplification of kDNA maxicircle targets. In addition, MCAM/ET/2013/009 is a natural akinetoplastic stock based on absence of kDNA minicircle amplification and loss of kinetoplast DAPI staining [41].

### Expansion of trypanosome populations in mice

Trypanosome cryostabilates were thawed in a water bath at 37°C for 5 min, mixed with an equal volume of phosphate buffered saline glucose (PSG; 7.5 g/l Na_2_HPO_4_.2H_2_O, 0.34 g/l NaH_2_PO_4_.H_2_O, 2.12 g/l NaCl, 10 g/l D-glucose, pH 8) and checked for viability and motility of trypanosomes using microscopy. Swiss albino female mice of 6–8 weeks old and weighing between 25 and 30 g, obtained from the laboratory animal facility of the College of Veterinary Medicine of Mekelle University, were inoculated intraperitoneally (IP) with 0.2 ml of the trypanosome suspension. The parasitemia was monitored following the Matching Method, i.e. 5 μl of blood was transferred onto a microscope slide, covered with a 24x24 mm cover slip, examined at 40x10 magnification and the number of parasites per field of view were estimated and converted to parasites per ml of blood [42]. At peak parasitaemia, the mice were anaesthetised and exsanguinated by heart puncture with a heparinised syringe. Blood was diluted in PSG to a concentration of 2 trypanosomes per field (about 8x10^7^ trypanosomes/ml) prior to use for *in vivo* drug sensitivity testing.

### *In vivo* drug sensitivity testing

Per experimental group, 6 mice were inoculated intraperitoneally (IP) with 2.5x10^7^ living trypanosomes in PSG. Infection of each animal was confirmed individually by microscopy one day before treatment. Treatment started at day 4 post-infection and consisted of daily IP injections for 4 consecutive days with 0.1 ml/10g body weight (BW) 0.9% NaCl saline solution containing the appropriate concentration of drug. Five trypanocidal drugs were tested in this study. Melarsamine hydrochloride (MelCy; Cymelarsan), isometamidium chloride hydrochloride (ISM; Veridium), and diminazene diaceturate (DIM; Veriben) were procured in Europe. Diminazene diaceturate plus phenazone (DIM-SEQ; Sequzene) and homidium chloride (HOM; Bovidium) were procured from the local market in Shire Endaselasse, Western zone of Tigray regional state. All drugs, except MelCy, were assessed by the Animal Products, Veterinary Drug and Feed Quality Assessment Center in Addis Ababa (Ethiopia) for adherence to the physiciochemical characteristics stated by their manufacturers. The scientific name, trade name, origin and dosage of the drugs are presented in Table 1.

**Table 1:** Scientific name, trade name, provider and dosage of the trypanocidal drugs used in this study.

Scientific name	Trade name	Provider	Dose (mg/kg)	Recommended dose in domestic animals (mg/kg)
Bis(aminoethylthio) 4 melaminophenylarsine dihydrochloride (MelCy)	Cymelarsan	Merial, Lyon, France	0.125	0.25 (Merial, instruction leaflet)
			2	
Isometamidium chloride hydrochloride (ISM)	Veridium	Ceva Santé Animale, Libourne, France	1	0.5 [43]
Diminazene diaceturate and antipyrine (DIM)	Veriben	Ceva Santé Animale, Libourne, France	20	7 [43]
Diminazene diaceturate and phenazone granules (DIM-SEQ)	Sequzene	Alivira, Animal Health Limited, India	20	7 [43]
Homidium chloride (HOM)	Bovidium	Kela, Hoogstraten, Belgium	1	1 [44,45]

We tested the following doses: 0.125 mg/kg BW and 2 mg/kg BW MelCy, 1 mg/kg BW ISM, 20 mg/kg BW DIM or DIM-SEQ and 1 mg/kg BW HOM [28,43,46]. The control group consisted of infected mice that received 0.2 ml of saline solution [43].

Two days after the last treatment and subsequently once a week until day 60 post- treatment, each mouse was examined with the Matching Method for the presence of parasites. To detect subpatent parasitaemia, survivor mice were immunosuppressed with cyclophosphamide at 200 mg/kg BW (Endoxan, Baxter, Lessines, Belgium) 25 days post-treatment [47]. Relapsing mice were euthanised. At day 60 post-treatment, all mice that remained negative in microscopy, were tested by the microhaematocrit centrifugation technique (mHCT, 4 tubes per mouse) [48]. If negative in mHCT, all surviving mice were euthanised and their blood was collected on heparin by heart puncture. The blood of all mice from each group was pooled and run over a mini Anion Exchange Centrifugation Technique (mAECT) column to detect subpatent parasitaemia [49]. If negative in mAECT, the mice were considered to be cured.

## Results

Detailed data on the outcome of the mice after infection and treatment are given in S1 Table. All infected mice treated with 0.9% saline (controls) died between the onset of treatment and two days after treatment. Table 2 shows the observed number of relapses and the average day after treatment that relapses occurred. MelCy at 0.125 mg/kg BW cured only 2 out of 6 mice infected with MCAM/ET/2013/004 (type A) and none of the mice infected with MCAM/ET/2013/014 (type B). Therefore, this dose was not administered to the mice infected with the two other stocks. MelCy at a higher dose (2 mg/kg BW) and DIM at 20 mg/kg BW cured all mice infected with any *T*. *evansi* stock. Treatment with DIM-SEQ at 20 mg/kg BW caused relapses for all *T*. *evansi* stocks. HOM at 1 mg/kg BW failed to cure any mouse infected with any *T*. *evansi* stock, while ISM at 1 mg/kg BW cured 4 of the 6 mice infected with MCAM/ET/2013/10 and none of the mice infected with the other stocks. No particular difference was apparent in parasitemia during pretreatment and relapse, between the *T*. *evansi* type A and type B stocks.

**Table 2:** Drug sensitivity profile of *T*. *evansi* type A and *T*. *evansi* type B stocks against MelCy, DIM, DIM-SEQ, ISM and HOM in mice.

*T*. *evansi* stock, number of passages in mice and type	Drug (dosage)	Relapsed/ Treated	Average day post treatment of relapses occurring
MCAM/ET/2013/004	MelCy (2 mg/kg BW)	0/6	na
passage 5, type A	MelCy (0.125 mg/kg BW)	4/6	37.0
	DIM (20 mg/kg BW)	0/5*	na
	DIM-SEQ (20 mg/kg BW)	3/6	32.3
	ISM (1mg/kg BW)	5/5*	7.6
	HOM (1 mg/kg BW)	5/5*	2
MCAM/ET/2013/009	MelCy (2 mg/kg BW)	0/6	na
passage 5, type A	DIM (20 mg/kg BW)	0/6	na
	DIM-SEQ (20 mg/kg BW)	5/6	30.0
	ISM (1 mg/kg BW)	5/5*	3.4
	HOM (1 mg/kg BW)	5/5*	2
MCAM/ET/2013/010	MelCy (2 mg/kg BW)	0/6	na
passage 5, type B	DIM (20 mg/kg BW)	0/6	na
	DIM-SEQ (20 mg/kg BW)	4/5*	21.3
	ISM (1mg/kg BW)	2/6	12.5
	HOM (1 mg/kg BW)	5/5*	2
MCAM/ET/2013/014	MelCy (2 mg/kg BW)	0/6	na
passage 5, type B	MelCy (0.125 mg/kg BW)	6/6	7.6
	DIM (20 mg/kg BW)	0/6	na
	DIM-SEQ (20 mg/kg BW)	2/6	33.5
	ISM (1mg/kg BW)	6/6	13.2
	HOM (1 mg/kg BW)	6/6	2

* = one mouse died during treatment; na = not applicable

## Discussion

This experimental study was conducted with the objective to determine the *in vivo* drug sensitivity profile in a mouse model of some recently isolated *T*. *evansi* stocks from Ethiopia. We performed the single-dose test, using the recommended dosages of DIM and ISM to discriminate resistant from sensitive strains, as described by Eisler et al [43], yet we extended the treatment regimen from 1 to 4 consecutive daily administrations to increase drug availability. The experiment terminated after a 60 days follow-up period, including immunosuppression on day 25 after treatment to reveal cryptic ongoing infections that may otherwise remain undetectable [46,47]. This long follow-up is necessary since relapses may occur after one month. Unfortunately, some mice died before the end of treatment, demonstrating the high virulence of some *T*. *evansi* stocks and the inefficacy of the drug used. For further studies with these *T*. *evansi* stocks, lower infection doses or earlier start of treatment should be considered. Also, for follow-up we recommend to use more sensitive tests than the Matching Method, such the mHCT, which was used only after 60 days of follow-up, or even PCR. As we performed a single-dose test to measure resistance or sensitivity only once for each drug and *T*. *evansi* strain, future *in vivo* drug sensitivity tests should apply a multi-dose test to more accurately define the level of resistance of each isolate.

Nevertheless, this is the first study to describe the *in vivo* drug sensitivity of Ethiopian *T*. *evansi* stocks. Specifically, in our study we tested two stocks of the common *T*. *evansi* type A, and two stocks of the elusive *T*. *evansi* type B, for which currently limited data are available on diagnosis, host range, clinical progress and treatment options.

In this study, we did not find evidence for arsenical or diamidine resistance in Ethiopian *T*. *evansi*. Both 2 mg/kg Cymelarsan and 20 mg/kg Veriben were able to cure all mice, infected with any *T*. *evansi* strain or subtype. However, more than half of the *T*. *evansi* infected mice, that were treated with Sequzene relapsed in the 4^th^ week post- treatment, i.e. within maximum 8 days post-immunosuppression. Importantly, drug quality analysis of the used batches of both compounds reported that the purity of the compound complied with the manufacturer’s specifications (S1 Fig and S2 Fig). We have no conclusive answers to what caused this variability in cure rate. Given the fact that Veriben cured all *T*. *evansi* infected mice, it is unlikely that the difference with Sequzene can be attributed to diminazene resistance. Nevertheless, the Ethiopian stocks appear far less sensitive to cymelarsan and diminazene than reported for the Chinese isolate STIB 806K, where cure was obtained with < 0.125 mg/kg cymelarsan and with 2 mg/kg diminazene [46]. Previously, we showed that all Ethiopian *T*. *evansi* stocks used in this study appeared sensitive to cymelarsan and diminazene in *in vitro* drug testing [41]. Furthermore, all tested stocks carry a wild-type *TevAT1* sequence, which encodes in *T*. *evansi*, *T*. *equiperdum* and in *T*. *brucei* for an aminopurine transporter (P2) known to import diminazene and MelCy [41,50–54]. Arsenical, but not diminazene resistance, can also originate from mutations in the *TbAQP2-AQP3* locus, by either deletion or chimerisation of *TbAQP2* with *TbAQP3*, leading to reduced uptake of pentamidine and, to a lesser extent, of melarsen oxide [55–57]. However, considering the susceptibility of the Ethiopian *T*. *evansi* to cymelarsan, this genetic locus was not further explored in this study.

Veridium (ISM) and Bovidium (HOM) at 1.0 mg/kg failed to cure completely any *T*. *evansi* infection in mice. Drug quality analysis of the used batches of Veridium and Bovidium indicated that the purity of the compounds complied with the manufacturer’s specifications (S3 Fig and S4 Fig). Both drugs belong to the phenanthridine class of trypanocidal agents and both are assumed to accumulate in the kinetoplast, inhibiting transcription and replication of kDNA [58–60].

Recently, resistance to phenanthridines has been explained by genetic polymorphisms in the mitochondrial F1-ATPase y subunit, depletion of subunits of the vacuolar ATPase and absence of transport proteins that allow interaction between both ATPases [58,61,62]. Mutations in any of these proteins allow *T*. *brucei* to dispose of its kDNA, which in turn leads to phenanthridine and diamidine resistance [58,61,62]. Naturally dyskinetoplastic trypanosomes, such as *T*. *evansi* and *T*. *equiperdum*, have defined single nucleotide polymorphisms in the mitochondrial F1-ATPase y subunit that predispose them for complete loss of kDNA and thus cause innate resistance to primarily kDNA targeting drugs [63–65]. Interestingly, therapeutic failure of ISM in mice and rats infected with *T*. *evansi* stocks from Indonesia and Nigeria was observed by others [66,67]. Furthermore, ISM treatment failures of *T*. *evansi* infections were previously reported in Sudan, China, the Philippines and Venezuela [30,68,69]. All Ethiopian *T*.*evansi* stocks in this study have corresponding polymorphisms in the F1-ATPase y subunit for type A and type B [41,64,65,70]. While the F1-ATPase y subunit A281del mutation, which characterises *T*. *evansi* subtype A, could be clearly linked to dyskinetoplasty and ISM resistance by genetic studies in *T*. *brucei*, similar studies could not confirm the effect of the F1-ATPase y subunit M282L mutation, which characterises *T*. *evansi* subtype B [62]. In this report, we provide for the first time evidence that *T*. *evansi* type B, like *T*. *evansi* type A, are naturally *in vivo* resistant to the phenanthridine class of trypanocidal drugs, despite earlier evidence that both types can be killed *in vitro* by ISM [41]. Interestingly, for *T*. *evansi* there appears to be no correlation between *in vitro* and *in vivo* ISM sensitivity. This phenomenon was already noted decades ago [21].

### Conclusions

We conclude that Ethiopian *T*. *evansi* can be treated in mice by diminazene and MelCy regardless of *T*. *evansi* type and presence of kinetoplast. However, measures should be taken by the Ethiopian Veterinary Drug and Animal Feed Administration and Control Authority (VDAFACA) to create market access to Cymelarsan, which is currently not registered in Ethiopia, and to ensure consistent quality of commercial drug formulations that are available from the local markets. A recent study found that 27.3% of the diminaze diaceturate formulations and 29.4% of the isometamidium chloride formulations failed to comply with quality requirements as assessed in HPLC [71]. Furthermore, the phenanthridines isometamidium chloride and homidum salts are DNA intercalating agents that raise serious concerns of mutagenicity and are not well tolerated by camels [72]. Unfortunately, they are still in use for treating animals that provide beef and milk for human consumption [18,59,73].

## Supporting information

S1 Table Details on the outcome of mice infected with different *T*. *evansi* stocks and treated with different drugs. ISM = isometamidium chloride hydrochloride, DIM = diminazene diaceturate, DIM-SEQ = diminazene diaceturate and phenazone granules, MelCy = melarsamine hydrochloride, HOM = homidium chloride. D = death, N = no parasites detected in blood, P = parasites detected in blood, T = treatment.
(DOCX)

S1 Fig Physicochemical test result of the used batch of Veriben. (PDF)

S2 Fig Physicochemical test result of the used batch of Sequzene. (PDF)

S3 Fig Physicochemical test result of the used batch of Veridium. (PDF)

S4 Fig Physicochemical test result of the used batch of Bovidium. (PDF)
