# Supplementary material for: Isometamidium chloride and homidium chloride fail to cure mice infected with Ethiopian Trypanosoma evansi type A and B
Source: PLoS Negl Trop Dis. 2018 Sep 12;12(9):e0006790. doi: 10.1371/journal.pntd.0006790 (PMC6152993; doi:10.1371/journal.pntd.0006790)
Supplement: S1 Table — ISM = isometamidium chloride hydrochloride, DIM = diminazene diaceturate, DIM-SEQ = diminazene diaceturate and phenazone granules, MelCy = melarsamine hydrochloride, HOM = homidium chloride. D = death, N = no parasites detected in blood, P = parasites detected in blood, T = treatment. (DOCX) [file pntd.0006790.s001.docx]

**Supplementary Table S1.** Details on the outcome of mice infected with different *T. evansi* stocks and treated with different drugs.

ISM = isometamidium chloride hydrochloride, DIM = diminazene diaceturate, DIM-SEQ = diminazene diaceturate and phenazone granules, MelCy = melarsamine hydrochloride, HOM = homidium chloride.

D = death, N = no parasites detected in blood, P = parasites detected in blood, T = treatment

|  |  |  | **Day post infection** | | | | | | | | | | | | | |
| --- | --- | --- | --- | --- | --- | --- | --- | --- | --- | --- | --- | --- | --- | --- | --- | --- |
| ***T. evansi* stock** | **Drug and dosage** | **Mouse ID** | **3** | **4** | **5** | **6** | **7** | **9** | **16** | **23** | **30** | **37** | **44** | **51** | **58** | **67** |
| MCAM/ET/2013/004 | 0.9% saline | 1 | P | T | D |  |  |  |  |  |  |  |  |  |  |  |
|  |  | 2 | P | T | T | T | D |  |  |  |  |  |  |  |  |  |
|  |  | 3 | P | T | D |  |  |  |  |  |  |  |  |  |  |  |
|  |  | 4 | P | T | T | T | T | D |  |  |  |  |  |  |  |  |
|  |  | 5 | P | T | T | T | D |  |  |  |  |  |  |  |  |  |
|  |  | 6 | P | T | D |  |  |  |  |  |  |  |  |  |  |  |
|  | MelCy | 1 | P | T | T | T | T | N | N | N | N | N | D |  |  |  |
|  | 0.125 mg/kg BW | 2 | P | T | T | T | T | N | N | N | N | N | D |  |  |  |
|  |  | 3 | P | T | T | T | T | N | N | N | N | N | P |  |  |  |
|  |  | 4 | P | T | T | T | T | N | N | N | N | N | P |  |  |  |
|  |  | 5 | P | T | T | T | T | N | N | N | N | N | N | N | N | N |
|  |  | 6 | P | T | T | T | T | N | N | N | N | N | N | N | N | N |
|  | MelCy | 1 | P | T | T | T | T | N | N | N | N | N | N | N | N | N |
|  | 2 mg/kg BW | 2 | P | T | T | T | T | N | N | N | N | N | N | N | N | N |
|  |  | 3 | P | T | T | T | T | N | N | N | N | N | N | N | N | N |
|  |  | 4 | P | T | T | T | T | N | N | N | N | N | N | N | N | N |
|  |  | 5 | P | T | T | T | T | N | N | N | N | N | N | N | N | N |
|  |  | 6 | P | T | T | T | T | N | N | N | N | N | N | N | N | N |
|  | ISM | 1 | P | T | T | T | T | N | P |  |  |  |  |  |  |  |
|  | 1 mg/kg BW | 2 | P | T | T | T | T | P |  |  |  |  |  |  |  |  |
|  |  | 3 | P | T | T | T | T | N | P |  |  |  |  |  |  |  |
|  |  | 4 | P | T | D |  |  |  |  |  |  |  |  |  |  |  |
|  |  | 5 | P | T | T | T | T | N | P |  |  |  |  |  |  |  |
|  |  | 6 | P | T | T | T | T | N | D |  |  |  |  |  |  |  |
|  | DIM | 1 | P | T | T | T | T | N | N | N | N | N | N | N | N | N |
|  | 20 mg/kg BW | 2 | P | T | T | T | T | N | N | N | N | N | N | N | N | N |
|  |  | 3 | P | T | T | T | D |  |  |  |  |  |  |  |  |  |
|  |  | 4 | P | T | T | T | T | N | N | N | N | N | N | N | N | N |
|  |  | 5 | P | T | T | T | T | N | N | N | N | N | N | N | N | N |
|  |  | 6 | P | T | T | T | T | N | N | N | N | N | N | N | N | N |
|  | DIM-SEQ | 1 | P | T | T | T | T | N | N | N | N | N | N | N | N | N |
|  | 20 mg/kg BW | 2 | P | T | T | T | T | N | N | N | N | N | N | N | N | N |
|  |  | 3 | P | T | T | T | T | N | N | N | N | D |  |  |  |  |
|  |  | 4 | P | T | T | T | T | N | N | N | N | P |  |  |  |  |
|  |  | 5 | P | T | T | T | T | N | N | N | N | N | N | N | N | N |
|  |  | 6 | P | T | T | T | T | N | N | N | N | N | P |  |  |  |
|  | HOM | 1 | P | T | T | T | T | P |  |  |  |  |  |  |  |  |
|  | 1 mg/kg BW | 2 | P | T | T | T | T | P |  |  |  |  |  |  |  |  |
|  |  | 3 | P | T | T | T | T | P |  |  |  |  |  |  |  |  |
|  |  | 4 | P | T | T | T | D |  |  |  |  |  |  |  |  |  |
|  |  | 5 | P | T | T | T | T | P |  |  |  |  |  |  |  |  |
|  |  | 6 | P | T | T | T | T | P |  |  |  |  |  |  |  |  |
| MCAM/ET/2013/009 | 0.9% saline | 1 | P | T | T | T | D |  |  |  |  |  |  |  |  |  |
|  |  | 2 | P | T | D |  |  |  |  |  |  |  |  |  |  |  |
|  |  | 3 | P | D |  |  |  |  |  |  |  |  |  |  |  |  |
|  |  | 4 | P | T | T | D |  |  |  |  |  |  |  |  |  |  |
|  |  | 5 | P | T | D |  |  |  |  |  |  |  |  |  |  |  |
|  |  | 6 | P | T | T | D |  |  |  |  |  |  |  |  |  |  |
|  | MelCy | 1 | P | T | T | T | T | N | N | N | N | N | N | N | N | N |
|  | 2 mg/kg BW | 2 | P | T | T | T | T | N | N | N | N | N | N | N | N | N |
|  |  | 3 | P | T | T | T | T | N | N | N | N | N | N | N | N | N |
|  |  | 4 | P | T | T | T | T | N | N | N | N | N | N | N | N | N |
|  |  | 5 | P | T | T | T | T | N | N | N | N | N | N | N | N | N |
|  |  | 6 | P | T | T | T | T | N | N | N | N | N | N | N | N | N |
|  | ISM | 1 | P | T | T | T | D |  |  |  |  |  |  |  |  |  |
|  | 1 mg/kg BW | 2 | P | T | T | T | T | P |  |  |  |  |  |  |  |  |
|  |  | 3 | P | T | T | T | T | N | P |  |  |  |  |  |  |  |
|  |  | 4 | P | T | T | T | T | P |  |  |  |  |  |  |  |  |
|  |  | 5 | P | T | T | T | T | P |  |  |  |  |  |  |  |  |
|  |  | 6 | P | T | T | T | T | P |  |  |  |  |  |  |  |  |
|  | DIM | 1 | P | T | T | T | T | N | N | N | N | N | N | N | N | N |
|  | 20 mg/kg BW | 2 | P | T | T | T | T | N | N | N | N | N | N | N | N | N |
|  |  | 3 | P | T | T | T | T | N | N | N | N | N | N | N | N | N |
|  |  | 4 | P | T | T | T | T | N | N | N | N | N | N | N | N | N |
|  |  | 5 | P | T | T | T | T | N | N | N | N | N | N | N | N | N |
|  |  | 6 | P | T | T | T | T | N | N | N | N | N | N | N | N | N |
|  | DIM-SEQ | 1 | P | T | T | T | T | N | N | N | N | P |  |  |  |  |
|  | 20 mg/kg BW | 2 | P | T | T | T | T | N | N | N | D |  |  |  |  |  |
|  |  | 3 | P | T | T | T | T | N | N | N | N | P |  |  |  |  |
|  |  | 4 | P | T | T | T | T | N | N | N | N | P |  |  |  |  |
|  |  | 5 | P | T | T | T | T | N | N | N | N | N | N | N | N | N |
|  |  | 6 | P | T | T | T | T | N | N | N | N | N | P |  |  |  |
|  | HOM | 1 | P | T | T | T | T | P |  |  |  |  |  |  |  |  |
|  | 1 mg/kg BW | 2 | P | T | T | T | D |  |  |  |  |  |  |  |  |  |
|  |  | 3 | P | T | T | T | T | P |  |  |  |  |  |  |  |  |
|  |  | 4 | P | T | T | T | T | P |  |  |  |  |  |  |  |  |
|  |  | 5 | P | T | T | T | T | P |  |  |  |  |  |  |  |  |
|  |  | 6 | P | T | T | T | T | P |  |  |  |  |  |  |  |  |
| MCAM/ET/2013/010 | 0.9% saline | 1 | P | T | D |  |  |  |  |  |  |  |  |  |  |  |
|  |  | 2 | P | T | T | D |  |  |  |  |  |  |  |  |  |  |
|  |  | 3 | P | T | T | T | D |  |  |  |  |  |  |  |  |  |
|  |  | 4 | P | T | T | T | T | D |  |  |  |  |  |  |  |  |
|  |  | 5 | P | D |  |  |  |  |  |  |  |  |  |  |  |  |
|  |  | 6 | P | T | D |  |  |  |  |  |  |  |  |  |  |  |
|  | MelCy | 1 | P | T | T | T | T | N | N | N | N | N | N | N | N | N |
|  | 2 mg/kg BW | 2 | P | T | T | T | T | N | N | N | N | N | N | N | N | N |
|  |  | 3 | P | T | T | T | T | N | N | N | N | N | N | N | N | N |
|  |  | 4 | P | T | T | T | T | N | N | N | N | N | N | N | N | N |
|  |  | 5 | P | T | T | T | T | N | N | N | N | N | N | N | N | N |
|  |  | 6 | P | T | T | T | T | N | N | N | N | N | N | N | N | N |
|  | ISM | 1 | P | T | T | T | T | N | N | N | N | N | N | N | N | N |
|  | 1 mg/kg BW | 2 | P | T | T | T | T | N | N | N | N | N | N | N | N | N |
|  |  | 3 | P | T | T | T | T | N | N | N | N | N | N | N | N | N |
|  |  | 4 | P | T | T | T | T | N | N | P |  |  |  |  |  |  |
|  |  | 5 | P | T | T | T | T | N | N | N | N | N | N | N | N | N |
|  |  | 6 | P | T | T | T | T | N | P |  |  |  |  |  |  |  |
|  | DIM | 1 | P | T | T | T | T | N | N | N | N | N | N | N | N | N |
|  | 20 mg/kg BW | 2 | P | T | T | T | T | N | N | N | N | N | N | N | N | N |
|  |  | 3 | P | T | T | T | T | N | N | N | N | N | N | N | N | N |
|  |  | 4 | P | T | T | T | T | N | N | N | N | N | N | N | N | N |
|  |  | 5 | P | T | T | T | T | N | N | N | N | N | N | N | N | N |
|  |  | 6 | P | T | T | T | T | N | N | N | N | N | N | N | N | N |
|  | DIM-SEQ | 1 | P | T | T | T | T | N | N | N | P |  |  |  |  |  |
|  | 20 mg/kg BW | 2 | P | T | T | T | T | N | N | N | N | N | P |  |  |  |
|  |  | 3 | P | T | T | T | T | N | N | N | N | N | N | N | N | N |
|  |  | 4 | P | T | T | T | T | N | D |  |  |  |  |  |  |  |
|  |  | 5 | P | T | T | T | T | N | N | P |  |  |  |  |  |  |
|  |  | 6 | P | T | T | D |  |  |  |  |  |  |  |  |  |  |
|  | HOM | 1 | P | T | T | T | D |  |  |  |  |  |  |  |  |  |
|  | 1 mg/kg BW | 2 | P | T | T | T | T | P |  |  |  |  |  |  |  |  |
|  |  | 3 | P | T | T | T | T | P |  |  |  |  |  |  |  |  |
|  |  | 4 | P | T | T | T | T | P |  |  |  |  |  |  |  |  |
|  |  | 5 | P | T | T | T | T | P |  |  |  |  |  |  |  |  |
|  |  | 6 | P | T | T | T | T | P |  |  |  |  |  |  |  |  |
| MCAM/ET/2013/14 | 0.9% saline | 1 | P | T | T | T | T | D |  |  |  |  |  |  |  |  |
|  |  | 2 | P | T | T | D |  |  |  |  |  |  |  |  |  |  |
|  |  | 3 | P | T | T | T | D |  |  |  |  |  |  |  |  |  |
|  |  | 4 | P | T | T | D |  |  |  |  |  |  |  |  |  |  |
|  |  | 5 | P | T | D |  |  |  |  |  |  |  |  |  |  |  |
|  |  | 6 | P | T | T | T | D |  |  |  |  |  |  |  |  |  |
|  | MelCy | 1 | P | T | T | T | T | N | D |  |  |  |  |  |  |  |
|  | 0.125 mg/kg BW | 2 | P | T | T | T | T | N | P |  |  |  |  |  |  |  |
|  |  | 3 | P | T | T | T | T | N | P |  |  |  |  |  |  |  |
|  |  | 4 | P | T | T | T | T | N | P |  |  |  |  |  |  |  |
|  |  | 5 | P | T | T | T | T | P |  |  |  |  |  |  |  |  |
|  |  | 6 | P | T | T | T | T | N | P |  |  |  |  |  |  |  |
|  | MelCy | 1 | P | T | T | T | T | N | N | N | N | N | N | N | N | N |
|  | 2 mg/kg BW | 2 | P | T | T | T | T | N | N | N | N | N | N | N | N | N |
|  |  | 3 | P | T | T | T | T | N | N | N | N | N | N | N | N | N |
|  |  | 4 | P | T | T | T | T | N | N | N | N | N | N | N | N | N |
|  |  | 5 | P | T | T | T | T | N | N | N | N | N | N | N | N | N |
|  |  | 6 | P | T | T | T | T | N | N | N | N | N | N | N | N | N |
|  | ISM | 1 | P | T | T | T | T | N | P |  |  |  |  |  |  |  |
|  | 1 mg/kg BW | 2 | P | T | T | T | T | N | P |  |  |  |  |  |  |  |
|  |  | 3 | P | T | T | T | T | N | P |  |  |  |  |  |  |  |
|  |  | 4 | P | T | T | T | T | N | N | N | P |  |  |  |  |  |
|  |  | 5 | P | T | T | T | T | N | N | D |  |  |  |  |  |  |
|  |  | 6 | P | T | T | T | T | N | P |  |  |  |  |  |  |  |
|  | DIM | 1 | P | T | T | T | T | N | N | N | N | N | N | N | N | N |
|  | 20 mg/kg BW | 2 | P | T | T | T | T | N | N | N | N | N | N | N | N | N |
|  |  | 3 | P | T | T | T | T | N | N | N | N | N | N | N | N | N |
|  |  | 4 | P | T | T | T | T | N | N | N | N | N | N | N | N | N |
|  |  | 5 | P | T | T | T | T | N | N | N | N | N | N | N | N | N |
|  |  | 6 | P | T | T | T | T | N | N | N | N | N | N | N | N | N |
|  | DIM-SEQ | 1 | P | T | T | T | T | N | N | N | N | D |  |  |  |  |
|  | 20 mg/kg BW | 2 | P | T | T | T | T | N | N | N | N | N | D |  |  |  |
|  |  | 3 | P | T | T | T | T | N | N | N | N | N | N | N | N | N |
|  |  | 4 | P | T | T | T | T | N | N | N | N | N | N | N | N | N |
|  |  | 5 | P | T | T | T | T | N | N | N | N | N | N | N | N | N |
|  |  | 6 | P | T | T | T | T | N | N | N | N | N | N | N | N | N |
|  | HOM | 1 | P | T | T | T | T | P |  |  |  |  |  |  |  |  |
|  | 1 mg/kg BW | 2 | P | T | T | T | T | P |  |  |  |  |  |  |  |  |
|  |  | 3 | P | T | T | T | T | P |  |  |  |  |  |  |  |  |
|  |  | 4 | P | T | T | T | T | P |  |  |  |  |  |  |  |  |
|  |  | 5 | P | T | T | T | T | P |  |  |  |  |  |  |  |  |
|  |  | 6 | P | T | T | T | T | P |  |  |  |  |  |  |  |  |
