# Supplementary material for: Isometamidium chloride and homidium chloride fail to cure mice infected with Ethiopian Trypanosoma evansi type A and B
Source: PLoS Negl Trop Dis. 2018 Sep 12;12(9):e0006790. doi: 10.1371/journal.pntd.0006790 (PMC6152993; doi:10.1371/journal.pntd.0006790)
Supplement: S1 Fig — (PDF) [file pntd.0006790.s002.pdf]

ANIMAL PRODUCTS, VETERINARY DRUG AND FEED QUALITY ASSESSMENT CENTRE
Tel:+251-114-71-79-64
+251-114-71-72-58
P.O.Box:31303
Addis Ababa, Ethiopia

MEKELLE UNIVERSITY
COLLEGE OF VETERINARY MEDICINE
Tel:+251-344-40-13-89
Fax:+251-344-40-15-95
P.O.Box:2084
Mekelle, Ethiopia

Certificate No.
QMS_DQA_444_12/06/2018

Drug physicochemical Certificate of Analysis (CoA) — **QMS_FORM_014**

1. SAMPLE INFORMATION

Sample submission date	May 31/2018	Sampling method	No information
Sample ID	QMS_DQA_444_2018	Customer Ref. No	CVM/19762/01
Brand Name	Veriben	Generic name	Diminazene diaceturate
Formulation	Granules for injection	Presentation	Sachet of 23.6g
Composition	Diminazene diaceturate 10.5g & Antipyrine 23.6g/sachet	Batch/Lot No.	830A1
Mfg. Date	Mar. /2015	Expiry Date	Mar. /2019
Manufacturer	Ceva Sante Animale	For the account of	Mekelle Universty Research
Submitted by	Birehanu Hadush(Dr.)	Method of analysis	Manufacturer
Analysis request date	Jun. 01/2018	Date report prepared	Jun. 12/2018

2. PHYSICOCHEMICAL TEST RESULTS

Analysis date	Test parameters	Specification/acceptance limit	Observation	Conclusion
Jun. 11/2018	Appearance	Yellow, granules powder	Yellow, granules powder	**Complies**
Jun. 11/2018	Identification by UV-Vis	UV-Vis spectrum of standard complies to the sample.	UV-Vis spectrum of standard complies with sample.	**Complies**
Jun. 11/2018	Assay/API determination by UV-Vis with Working Standard (WS)<br>Diminazene diaceturate<br>Antipyrine(Phenazone)	 90 to 110%<br>90 to 110%	 94.71%<br>93.11%	**Complies**

3. **GENERAL CONCLUSION :** The tested sample meets the requirements as per Manufacturer method.

4. **REMARK:** This report and its test results relates only to the specific sample (s) identified herein and do not apply to any similar item that has not been tested.

5. FINAL TEST RESULT AUTHORIZATION

Final test report	Name	Signature	Date
**Reviewed by**	Tadese Setegn	[signature]	Jun. 12/2018
**Assured by**	Ayalew Zelelew for/Director Laboratory Quality Management Control Directorate	[signature]	Jun. 12/2018
**Authorized by**	Belachew Tefera Zerihun (Dr)	[signature]	Jun. 12/2018

Manager
Animal products, Veterinary Drug & Feed Quality Assessment Centre

CONTROLLED COPY
