# Supplementary material for: Isometamidium chloride and homidium chloride fail to cure mice infected with Ethiopian Trypanosoma evansi type A and B
Source: PLoS Negl Trop Dis. 2018 Sep 12;12(9):e0006790. doi: 10.1371/journal.pntd.0006790 (PMC6152993; doi:10.1371/journal.pntd.0006790)
Supplement: S2 Fig — (PDF) [file pntd.0006790.s003.pdf]

	**ANIMAL PRODUCTS, VETERINARY DRUG AND FEED QUALITY ASSESSMENT CENTRE**	**MEKELLE UNIVERSITY COLLEGE OF VETERINARY MEDICINE**	**Certificate No. QMS_DQA_447_12/06/2018**
	Tel: + 251-114-71-79-64 / 011-4-71-72-58<br>P.O.Box: 31303<br>Addis Ababa, Ethiopia	Tel: +251-344-40-13-89<br>Fax: +251-344-40-15-95<br>P.O. Box : 2084<br>Mekelle, Ethiopia	
	**Drug physicochemical Certificate of Analysis (CoA)**		**QMS_FORM_014**

1. SAMPLE INFORMATION

Sample submission date	May 31/2018	Sampling method	Random
Sample ID	QMS_DQA_447_2018	Customer Ref. No	CVM/19762/01
Brand Name	SEQUZENE	Generic name	Diminazene diaceturate
Formulation	Granules for injection	Presentation	Sachet of 2.36g
Composition	Diminazene diaceturate 1.05g & Phenazone (Antipyrine) 1.31g/sachet	Batch/Lot No.	GR/15014/01
Mfg. Date	Sep. 2015	Expiry Date	Apr. /2020
Manufacturer	Alivira Animal Health Limited	For the account of	Mekelle University Research
Submitted by	Birehanu Hadush (Dr)	Method of analysis	Manufacturer
Analysis request date	Jun 01/2018	Date report prepared	Jun. 12/2018

2. PHYSICOCHEMICAL TEST RESULTS

Analysis date	Test parameters	Specification/acceptance limit	Observation	Conclusion
Jun. 11/2018	Appearance	Yellow, granule powder	Yellow, granules powder	**Complies**
Jun. 11/2018	Identification by UV-Vis	UV-Vis spectrum of standard complies to the sample.	UV-Vis spectrum of standard complies with sample.	**Complies**
Jun. 11/2018	Assay/API determination by UV-Vis with working standard (WS)<br><br>Diminazene diaceturate<br>Antipyrine (phenazone)	 <br><br>90 to 110%<br>90 to 110%	 <br><br>95.21%<br>97.61%	**Complies**

1. **GENERAL CONCLUSION:** The tested sample **meets** the requirements as per Manufacturer Method.
2. **REMARK:** the report and its test results relates only to the specific sample(s) identified herein and onot apply to any similar item that has not been tested.

3. FINAL TEST RESULT AUTHORIZATION

Final test report	Name	Signature	Date
**Reviewed by**	Tadese Setegn	[signature]	Jun 12/2018
**Assured by**	Zerihun Abegaz Yassin (Dr)<br>Director<br>Laboratory Quality Management<br>Control Directorate	[signature]	Jun 12/2018
**Authorized by**	Belachew Tefera Zerihun (Dr)<br>**APVD-FQAC Quality Assurance**<br>Manager<br>Animal products, Veterinary Drug &<br>Feed Quality Assessment Centre	[signature]	Jun 12/2018
