# Supplementary material for: Isometamidium chloride and homidium chloride fail to cure mice infected with Ethiopian Trypanosoma evansi type A and B
Source: PLoS Negl Trop Dis. 2018 Sep 12;12(9):e0006790. doi: 10.1371/journal.pntd.0006790 (PMC6152993; doi:10.1371/journal.pntd.0006790)
Supplement: S3 Fig — (PDF) [file pntd.0006790.s004.pdf]

	ANIMAL PRODUCTS, VETERINARY DRUG AND FEED QUALITY ASSESSMENT CENTRE<br>Tel:+251-114-71-79-64<br>+251-114-71-72-58<br>P.O.Box:31303<br>Addis Ababa, Ethiopia	MEKELLE UNIVERSITY<br>COLLEGE OF VETERINARY MEDICINE<br>Tel:+251-344-40-13-89<br>Fax:+251-344-40-15-95<br>P.O.Box:2084<br>Mekelle, Ethiopia	Certificate No.<br>QMS_DQA_445_12/06/2018
	Drug physicochemical Certificate of Analysis (CoA)		QMS_FORM_014

1. SAMPLE INFORMATION

Sample submission date	May 31/2018	Sampling method	No information
Sample ID	QMS_DQA_445_2018	Customer Ref. No	CVM/19762/01
Brand Name	Veridium	Generic name	Isometamidium chloride hydrochloride
Formulation	Powder for solution for injection	Presentation	Sachet of 1g
Composition	Isometamidium chloride hydrochloride 1g/sachet	Batch/Lot No.	245A1
Mfg. Date	Jan. /2015	Expiry Date	Jan. /2020
Manufacturer	Ceva Sante Animale	For the account of	Mekelle Universty Research
Submitted by	Birehanu Hadush(Dr.)	Method of analysis	Manufacturer
Analysis request date	Jun. 06/2018	Date report prepared	Jun. 12/2018

2. PHYSICOCHEMICAL TEST RESULTS

Analysis date	Test parameters	Specification/acceptance limit	Observation	Conclusion
Jun. 07/2018	Physical description	Dark red crystalline powder .	Dark red crystalline powder .	**Complies**
Jun. 07/2018	Identification test	Sample and Reference Material (RM) retention time should be the same.	Sample retention time is the same as that of Reference Material (RM).	**Complies**
Jun. 07/2018	Assay/API content Isometamidium chloride hydrochloride with Working Standard (WS)	90% to 110% labeled amount	99.80%	**Complies**

3. **GENERAL CONCLUSION :** The tested sample meets the requirements as per Manufacturer method.

4. **REMARK:** This report and its test results relates only to the specific sample (s) identified herein and do not apply to any similar item that has not been tested.

5. FINAL TEST RESULT AUTHORIZATION

Final test report	Name	Signature	Date
**Reviewed by**	Tadese Setegn	[signature]	Jun. 12/2018
**Assured by**	Ayalew Zelelew for/ Director Laboratory Quality Management Control Directorate	[signature]	Jun. 12/2018
**Authorized by**	Belachew Tefera Zerihun (Dr)	[signature]	Jun. 12/2018

Manager
Animal products, Veterinary Drug & Feed Quality Assessment Centre

CONTROLLED COPY
