# Supplementary material for: Isometamidium chloride and homidium chloride fail to cure mice infected with Ethiopian Trypanosoma evansi type A and B
Source: PLoS Negl Trop Dis. 2018 Sep 12;12(9):e0006790. doi: 10.1371/journal.pntd.0006790 (PMC6152993; doi:10.1371/journal.pntd.0006790)
Supplement: S4 Fig — (PDF) [file pntd.0006790.s005.pdf]

	ANIMAL PRODUCTS, VETERINARY DRUG AND FEED QUALITY ASSESSMENT CENTRE	MEKELLE UNIVERSITY COLLEGE OF VETERINARY MEDICINE	Certificate No. QMS_DQA_448_04/07/2018
	Tel: + 251-114-71-79-64 / 011-4-71-72-58<br>P.O.Box: 31303<br>Addis Ababa, Ethiopia	Tel: + 251-034-44-013-89<br>P.O.Box: 2084<br>Mekelle, Ethiopia	

DRUG PHYSICOCHEMICAL TEST RESULT REPORTING FORM

QMS_FORM_014

1. SAMPLE INFORMATION

Sample submission date	May 31/2018	Sampling method	Random
Sample ID	QMS_DQA_448_2018	Customer Ref. No	CVM/19762/01
Brand Name	Bovidium	Generic name	Homidium chloride
Formulation	Tablet for injection	Presentation	Tablet of 250mg
Composition	Homidium chloride 250mg/ tablet	Batch/Lot No.	20311
Mfg. Date	Jul. /2013	Expiry Date	Jun. /2018
Manufacturer	KELA N.V.	For the account of	MU / Shire endaslase vet pharmacy
Submitted by	Mekelle University	Method of analysis	Manufacturer
Analysis request date	Jun 27/2018	Date report prepared	04/07/2018

2. PHYSICOCHEMICAL TEST RESULTS

Analysis date	Test parameters	Specification/acceptance limit	Observation	Conclusion
03/07/2018	Appearance	Reddish purple, plain, biconvex round tablet	Reddish purple, plain, biconvex round tablet.	**Complies**
03/07/2018	Identification test by IR	The spectrum of the tablet complies with the IR reference spectrum.	The spectrum of the tablet complies with the IR reference spectrum.	**Complies**
03/07/2018	Assay<br>Homidium Bromide	90.0-110.0%<br>225.0-275.0 mg/tablet	96.09%<br>240.23 mg/tab	**Complies**

3. **GENERAL CONCLUSION:** The tested sample **meets** the requirements as per Manufacturer Methods.

4. **REMARK:** The test result is based on the test parameters carried out on the samples submitted to the lab by M.U.

5. FINAL TEST RESULT AUTHORIZATION

Final test report	Name	Signature	Date
**Reviewed by**	Tadese Setegn		**04/07/2018**
**Assured by**	Zerihun Abegaz Yassin (Dr)<br>Director<br>Laboratory Quality Management Control Directorate		**04/07/2018**
**Authorized by**	Belachew Tefera Zerihun (Dr)<br>Manager<br>Animal products,Veterinary Drug & Feed Quality Assessment Centre		**04/07/2018**

CONTROLLED COPY
